# From macro to micro: slow-wave sleep and its pivotal health implications

**DOI:** 10.3389/frsle.2024.1322995

**Published:** 2024-07-02

**Authors:** Toru Ishii, Pahnwat Tonya Taweesedt, Christina F. Chick, Ruth O'Hara, Makoto Kawai

**Affiliations:** ^1^Department of Psychiatry and Behavioral Sciences, Stanford University School of Medicine, Stanford, CA, United States; ^2^Sierra Pacific Mental Illness Research Education and Clinical Centers (MIRECC), VA Palo Alto Health Care System, Palo Alto, CA, United States

**Keywords:** sleep, macroarchitecture, microarchitecture, slow wave sleep, slow wave activity, delta wave, slow oscillation

## Abstract

Research on slow-wave sleep (SWS) began almost a century ago, not long after the discovery of electroencephalography. From maintaining homeostasis to memory function, the pivotal role of SWS in health has been established. The elucidation of its mechanisms and functions is directly related to the fundamental question of why people sleep. This comprehensive review first summarizes the basic science of SWS from anatomical and physiological aspects. It describes the fundamental mechanisms and functions of SWS, including hormonal regulation, developmental changes in SWS across the lifespan, and associations between SWS and optimal physical, psychological, and cognitive functions. Next, the relationship between SWS and physical and mental disorders, for which increasing knowledge has accumulated in recent years, is discussed from both research and clinical perspectives. Conditions such as memory impairment, sleep-disordered breathing, neurodevelopmental disorders, and various psychiatric disorders are of concern. The relationship between SWS and the glymphatic system, which is responsible for waste clearance in the brain, has also been explored, highlighting the potential neuroprotective role of SWS. Finally, we discuss the future direction of the field regarding whether interventions in SWS can improve health. We also address the problem of the inconsistent definitions of SWS, slow-wave activity, and slow oscillations. This review emphasizes the importance of discussing SWS from both macro- and microarchitectural perspectives and highlights its potential clinical and research impacts. By reviewing these aspects, we aim to contribute to a deeper understanding of SWS and the future development of this research field.

## 1 Introduction

Slow waves were first characterized by Loomis et al. in the 1930s, shortly after Hans Berger invented electroencephalography (EEG) (Loomis et al., 1935). Loomis also provided the first definition of sleep stages (Loomis et al., 1937). Rechtschaffen and Kales (1968) developed sleep stage classifications, and the definition of slow-wave sleep (SWS) was modified by the American Academy of Sleep Medicine (AASM) in 2007 (Iber and American Academy of Sleep, 2007). Meanwhile, it was not until nearly half a century after Loomis' report that details of the physiological activity underlying slow waves were described. In a series of studies published in 1993, Mircea Steriade recorded the physiological activity underlying slow-wave activity (SWA) in cortical and thalamic networks using intracellular and EEG recordings (Steriade et al., 1993a,b,c).

The invention of the sleep-stage classification has contributed to the development of both basic sleep science and clinical sleep medicine. Findings based on the sleep stage classification (sleep macroarchitecture) enabled sleep to become the subject of science. Meanwhile, since Steriade's paper, studies on sleep microarchitecture have also tremendously contributed to developing the sleep research field. In recent years, the importance of slow oscillations (SO) and other sleep microarchitectures in basic science and clinical medicine has been increasingly reported. However, unfortunately, because of their separate development, the terms SWS, mainly meaning macroarchitecture, and SO/delta oscillation, generally meaning microarchitecture, have been used with ambiguous definitions, and different definitions have been used interchangeably between research and clinical fields and even within the fields (see Section 1.1). In addition, in recent years, the distinct roles of the SO and delta waves in memory function have been reported (Kim et al., 2019), and mixed definitions of these terms have become critical issues that could impede the development of this field.

Modifying what has already become a practice in each field is difficult. Furthermore, different definitions do not diminish the validity of previously published results. However, we must avoid treating results derived from different definitions as if they were the same when discussing research and clinical findings. Science and medicine are evolving, and new findings accumulate daily, while we search for a point of consensus on definitions. Herein, this review aims to organize the findings on the macro- and microarchitectures of SWS so that those involved in this field can share their understanding. Hence, we describe the findings in each section from the macro- and microperspectives of slow waves with careful attention to their definitions in each study. We discuss the basic science of SWS, followed by clinical science and practice. We also address the current problems in the field and discuss future prospects.

### 1.1 Definitions of SWS, SWA, delta waves, and SO

The definitions and classifications of sleep stages have evolved over time, driven by advancements in neuroscientific research. Each stage of sleep is defined using EEG, a traditional tool that offers crucial information regarding brain waves, including amplitude and frequency.

**Figure 1:**
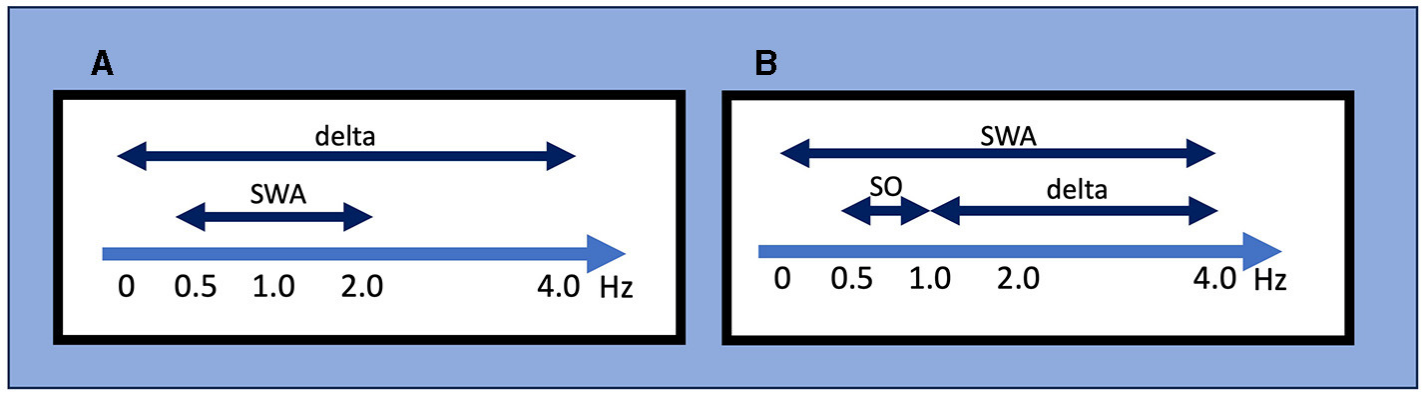
Definitions for slow-wave activity (SWA), slow oscillations (SO), and delta wave by different criteria**. (A)** The AASM criteria mainly used in the clinical setting. SWA is defined as a wave of frequency 0.5–2 Hz and peak-to-peak amplitude of >75 μV, and delta waves are defined as a rhythm consisting of 0–3.99 Hz activity. **(B)** An example of conventional criteria mainly used in the research setting. It should be noted that these frequency ranges used to define SWA, SO, and delta wave/oscillation often differ between studies. AASM, American Academy of Sleep Medicine; SO, slow oscillation; SWA, slow wave activity.

In their pioneer work, Loomis et al. divided sleep into five stages (from stage A to stage E) based on different EEG events (Loomis et al., 1937). Rechtschaffen and Kales (R&K) classified stage 3 and stage 4 sleep as epochs of sleep containing >20% and >50% of slow waves with frequencies <2 Hz and amplitudes >75 μV, respectively (Rechtschaffen and Kales, 1968). The 2007 AASM manual for scoring sleep and associated events replaced the R&K nomenclature of stages 3 and 4 sleep with non-rapid eye movement (NREM) stage 3 (N3) sleep. Stage N3 sleep is generally called SWS or “deep sleep,” and stage N3 sleep was scored when at least 20% of an epoch consisted of high-amplitude SWA (Iber and American Academy of Sleep, 2007). In this context, SWA is defined as a wave of frequency 0.5–2 Hz and peak-to-peak amplitude of >75 μV (Figure 1), measured by EEG over the frontal regions referenced to the contralateral ear or mastoid (F4-M1, F3-M2). Although the AASM manual refers to several acceptable derivations in addition to recommended ones (Troester et al., 2023), no criteria for SWA are defined with these acceptable derivations. Applying the above definition to the Fz–Cz montage from acceptable derivations should be avoided because of the shorter distance between Fz and Cz compared to F4-M1 or F3-M2, which affects the amplitude.

While this definition of SWS focuses on the sleep macroarchitecture, delta oscillation/SO is defined from the perspective of the sleep microarchitecture. Since the early days of EEG recording, researchers have named the oscillatory activity on the EEG according to its frequency range, with delta oscillation generally referring to 1–4-Hz waves (“delta oscillations” and “delta waves” are generally used interchangeably). In their landmark report, Steriade et al. (1993c) referred to waves <1 Hz as SO; to be exact, they also used the term “slow rhythm”. However, the frequency bands that define delta oscillation, SO, and SWA often differ among researchers (Figure 1). For instance, SWA is sometimes defined as EEG power <4 Hz but other times as power between 0.5 and 4.5 Hz. Moreover, the definition of SWA used in scoring SWS is specific to normal sleep architecture and may differ from SWA in other contexts. For example, although SWA is sometimes also called a “high amplitude delta,” SWA in clinical EEG interpretation refers to an activity with a frequency that falls within the delta frequency of 0–3.99 Hz without amplitude criteria and commonly indicates abnormal brain function in conditions such as diffuse slow activity in metabolic encephalopathy, epileptiform discharges in epilepsy, coma, and vegetative state (Frohlich et al., 2021).

In sum, different definitions of “slow waves” have been used in research and clinical fields, and different definitions exist even within each field (Figure 1). From historical sleep staging to contemporary updates, understanding the definition of SWS, SWA, and SO contributes to our comprehension of the broader landscape of sleep neurophysiology. We emphasize that recognizing the existence of different definitions is the starting point and that each study should be carefully examined based on this knowledge.

## 2 Basic science of SWS and SWA

### 2.1 Anatomy of SWS/SWA

#### 2.1.1 Origin and propagation of SWS

Steriade et al. (1993b) have reported that SOs persisted in the cortex even after the connected thalamus was destroyed, indicating that they originated from recurrent cortical connectivity (Timofeev and Steriade, 1996). SOs represent alternations in the neuronal network between synchronized membrane depolarization, known as “up-states,” and prolonged hyperpolarization, known as “down-states.” They exhibit non-stationary bistability even during the physical disconnection of the cerebral cortex.

Where in the cortex and how are slow waves generated? Although the cellular mechanisms underlying the generation of slow waves are still not fully understood, many important findings have been reported to date (Sanchez-Vives, 2020). *In vivo* and *in vitro* studies have consistently demonstrated that deep or infragranular layers, particularly the internal pyramidal cell layer of the cortex (layer V), play a crucial role in initiating up-states (Chauvette et al., 2010; Fiáth et al., 2016). During up-states, layer V neurons have more intense and longer discharge than layer II/III neurons (Capone et al., 2017). In addition, several studies have indicated that activation of layer V neurons by optogenetic stimulation can initiate up-states and induce SOs (Beltramo et al., 2013; Stroh et al., 2013).

Up-states may be triggered by the summation of miniature excitatory postsynaptic potentials (EPSPs) formed by the action-potential-independent release of transmitter vesicles (Timofeev et al., 2000). These EPSPs may originate from a residual synaptic activity resulting from stimulus processing during prior wakefulness and become sufficiently large to generate repetitive up-states, especially after intense learning. These intrinsic excitability in layer V neurons leads them to begin firing during down-states (Sanchez-Vives and McCormick, 2000; Compte et al., 2003; Sakata and Harris, 2009) and may enable them to function as a specific “pacemaker” (Le Bon-Jego and Yuste, 2007). After initiation, the up-state may be amplified by intrinsic currents such as persistent Na^+^ and high-threshold Ca^2+^ currents. Synchronization of activity during depolarizing states may be partially maintained via corticocortical glutamatergic synaptic connections. Since the early characterizations of SOs both *in vivo* and *in vitro*, a contribution of *N*-methyl-d-aspartate (NMDA) and α-amino-3-hydroxy-5-methyl-4-isoxazolepropionic acid (AMPA) receptors to establishing long-range synchrony has been reported (Steriade et al., 1993b; Sanchez-Vives and McCormick, 2000). The transition from the up- to down-state ends the synaptic reverberation that generates the up-state. The induction of this process has been mainly linked to a combination of outward K^+^ currents and disfacilitation resulting from the depression of excitatory synapses (Galarreta and Hestrin, 1998; Bazhenov et al., 2002; Benita et al., 2012). Different mechanisms involving K^+^ currents have been proposed, including adenosine triphosphate-dependent K^+^ currents (Cunningham et al., 2006), γ-aminobutyric acid (GABA)_B_ receptor-mediated responses (Perez-Zabalza et al., 2020), and Ca^2+^ and Na^+^-dependent K^+^ currents (Sanchez-Vives and McCormick, 2000). The up-state-to-down-state transition across distinct cortical regions suggests that subcortical input may synchronously facilitate the onset of the down-state (Volgushev et al., 2011; Sheroziya and Timofeev, 2014), and the thalamus has been considered to play a particularly important role by producing SOs in coordination with the cortex (Steriade et al., 1993b; Crunelli and Hughes, 2010). A recent study indicates the important role of the claustrum in providing global control of slow waves via cortical inhibitory cells (Narikiyo et al., 2020; Timofeev and Chauvette, 2020).

While SO refers to the temporal organization of the cortical activity, the term “slow wave” includes a spatial component because propagation is a fundamental property of waves. A study using source modeling of high-density EEG (hdEEG) recordings has reported that slow waves mostly originate from the center of the lateral sulci in the left hemisphere. Slow waves then mainly migrate from the anterior to the posterior brain regions, and the axis often involves cortical structures, such as the inferior frontal gyrus, anterior cingulate, posterior cingulate, and precuneus (Murphy et al., 2009). In addition, studies with other methodologies have also demonstrated that slow waves do not simply travel (Massimini et al., 2004), but they often do so preferentially through the major connectional backbone of the human cortex (Hagmann et al., 2008) and typically only involve a subset of brain areas (Nir et al., 2011). Thus, certain slow waves may be preferentially triggered by synapses that were recently strengthened during the wake period, priming specific circuits for consolidation (see Sections 2.1.2 and 2.2.1.1). Although these results have challenged several influential topographic observations of slow waves that have persisted since the original EEG recordings of sleep, they further posited the question of whether slow waves are local or global phenomena (see Section 2.1.2).

#### 2.1.2 Regulation of SWA

**Figure 2:**
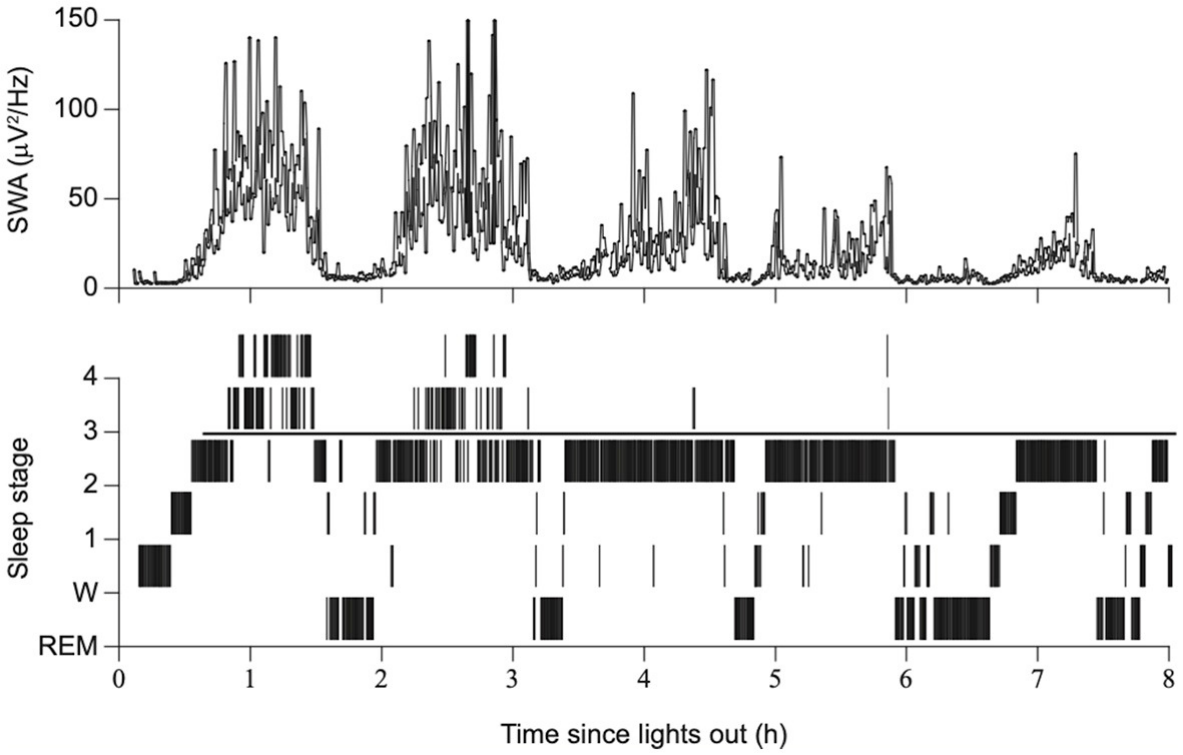
Sleep stages, slow wave activity, and time since lights out. The duration of SWS and SWA declines across consecutive NREM episodes overnight, with a gradual reduction in sleep drive. Modified from Dijk (2009).

**Figure 3:**
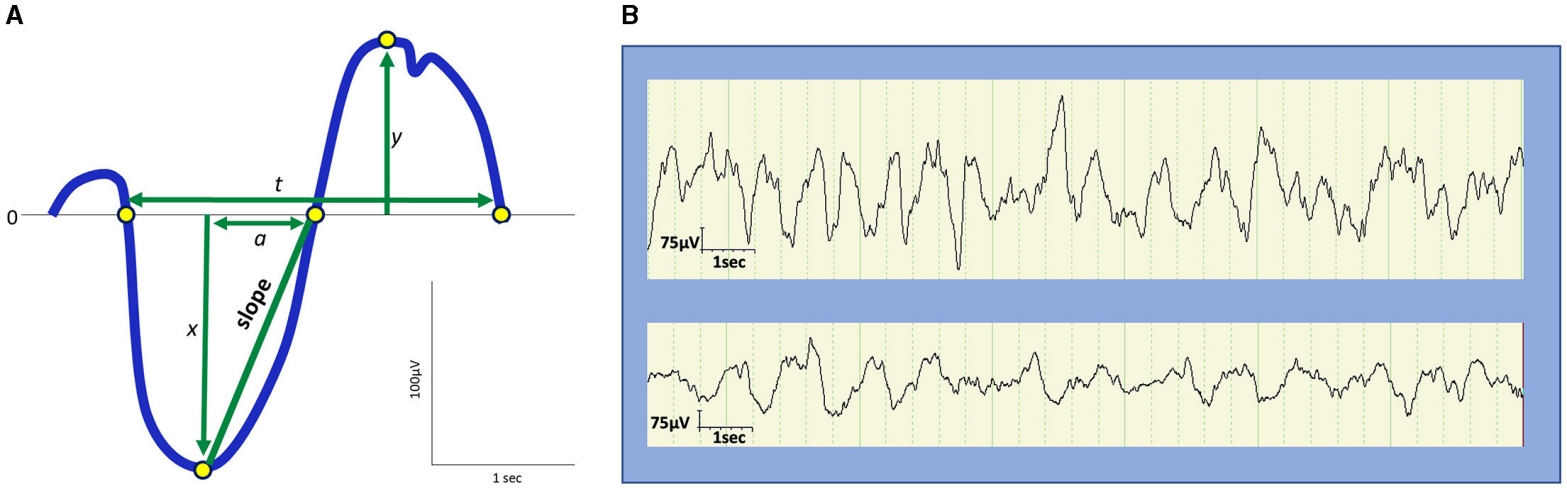
Amplitude, frequency, and slope of slow waves. **(A)** Schematic representation of slow waves. *x*, peak amplitude of an upward deflection; *y*, peak amplitude of a downward deflection; |*x*| + *y*, peak-to-peak amplitude; *t*, duration; 1/*t*, frequency (Hz); *a*, the duration from the negative peak to the next zero crossing. The slope is defined as |*x*|/*a*. **(B)** Examples of slow waves. Top: Slow waves in the first cycle stage N3 sleep during a night in a 9-year-old boy. Bottom: slow waves in stage N3 sleep in the latter half of a night in a 13-year-old boy. The top represents higher and steeper slow waves than the bottom.

In the early days of EEG, all waves were considered global phenomena from the viewpoint of macroarchitecture. However, after the aforementioned report that indicated SOs propagate, the local aspects of SO have been well recognized. Herein lies a shift in perspective from macro- to microarchitectures. Although the relative occurrence of local vs. global events remains debatable, their existence has been widely acknowledged (Nir et al., 2011; Vyazovskiy and Harris, 2013; Seok et al., 2022). Evidence has suggested that the SO in humans may be locally modified based on previous experiences. Specifically, the amplitude of the SO may be modulated by recent waking activities (Huber et al., 2004). This finding is closely linked to the synaptic homeostasis hypothesis (SHY) (Tononi and Cirelli, 2014) (see Section 2.2.1.1). This issue is directly related to what drives the SWS and how it is regulated. Although SWS and SWA are closely related to homeostatic regulation, several studies have reported that they are not influenced or regulated by the circadian clock (Dijk et al., 1990). Multiple studies have demonstrated that the amount of SWS and SWA in both humans and rodents is influenced by the time spent awake. Additionally, studies on healthy young individuals have indicated that SWA increases during the deeper stages of sleep and decreases as the night progresses (Achermann et al., 1993; Dijk, 1995, 2009) (Figure 2). Compared to the later part of the sleep, the early phase of sleep exhibits a steeper slope of slow waves, and the slopes were positively correlated with neuronal synchrony, indicating that neuronal firing patterns and slow wave slopes reflect the homeostatic control of SWS (Vyazovskiy et al., 2009) (Figure 3). Stage N3 sleep commonly predominates during the first half of the night. Regarding the characteristics of SWA in sleep stages, a very small amount of high-amplitude SWA can be observed during sleep in NREM stage 1 (N1), with SWA gradually increasing in NREM stage 2 (N2) and N3 and then returning to a very small amount in the rapid-eye movement (REM) sleep stage (Dijk, 2009).

Although the entire mechanism of SWS regulation remains to be elucidated, the quest for a mechanism is directly linked to the question of why SWS is necessary for organisms.

#### 2.1.3 Changes in SWS throughout the lifespan

The human brain develops at various stages of life. EEG during sleep has been suggested as a reliable method for monitoring age-related brain changes owing to its minimal susceptibility to confounding variables, such as environmental conditions during the day, thereby facilitating an unbiased evaluation of brain activity (Gorgoni et al., 2020). Exploring SWS throughout one's lifespan is essential for understanding dynamic shifts in sleep architecture and their potential impact on overall health.

SWA may be observed from the age of 2 months and commonly appears by the age of 4–5 months (Fattinger et al., 2014; Castelnovo et al., 2023). During early childhood, SWA tends to originate in more posterior regions of the brain than in adulthood (Timofeev et al., 2020). These changes in SWA topography progress from the posterior regions to the central area, and eventually to the frontal part of the brain in late adolescence (Castelnovo et al., 2023). In this study, the origin of SWA in SWS persisted in the frontal region throughout adulthood, possibly because of frontal cortex maturation. Although the same amplitude and frequency criteria for SWS are used in children and adults, slow waves in children display a larger amplitude (100–400 μV), steeper slope, and less widespread activity as compared to SWA in adults (Grigg-Damberger et al., 2007; Kurth et al., 2010) (Figure 3). Furthermore, SWA observed in children exhibits a greater tendency to originate from the right rather than the left hemisphere, potentially reflecting the incomplete development of interhemispheric white matter connections compared to that in adults (Castelnovo et al., 2023). SWS accounts for ~20%−30% of the total sleep time (TST) in children and 18%−22% of the TST in adults (Ohayon et al., 2004; McLaughlin Crabtree and Williams, 2009; Lee et al., 2022a). A meta-analysis of sleep parameters across the lifespan revealed that SWS comprises the largest percentage of sleep time in young children before puberty, diminishing with age at ~7% per 5 years from 5 to 15 years of age and 2% per 10 years in adulthood (Ohayon et al., 2004). However, a more recent meta-analysis that used only the 2007 AASM manual for scoring has revealed an age-related decline in TST without a significant age-related reduction in SWS (Boulos et al., 2019). Importantly, the former meta-analysis has also reported no significant change in SWS after 60 years of age. Meanwhile, a study based on two large cohort data with late-middle-aged and older adults has reported that delta power (1–4 Hz) decreases while slow power (<1 Hz) increases with age (Djonlagic et al., 2021a). The seemingly inconsistent reports on SWS/SWA changes in later life could be ascribed to different patterns of alterations between delta oscillations and SOs. When discussing changes in slow waves in older adults, being aware of the difference between sleep macro- and microarchitecture is particularly important.

The evolution of SWS characteristics throughout life, including amplitude, morphology, and regional origin, reflects the intricate maturation of the brain. Age-related SWS duration revealed in recent meta-analyses challenges conventional expectations, suggesting the need for further evidence.

#### 2.1.4 Microarchitectural analysis of slow waves

Initially, sleep analysis primarily focused on macroarchitecture, encompassing objective parameters assessed in a 30-s EEG epoch, such as sleep stages (N1, N2, SWS, and REM), sleep onset latency, and TST. Currently, the importance of sleep microarchitecture in elucidating sleep and its applications has been increasingly recognized. The analysis of sleep microarchitecture involves the quantification and evaluation of intra-state elements at a finer level by examining factors such as SO, delta power, and SWA. In contrast to macroarchitectural analysis, which evaluates sleep by stage, microarchitectural analysis enables researchers to evaluate sleep across stages or during a short nap. Various analysis methods, such as power spectrum analysis, connectivity analysis (see Section 2.1.5), and phase–amplitude coupling analysis (Cohen, 2008; Tort et al., 2010; Hyafil et al., 2015), have been widely used.

**Figure 4:**
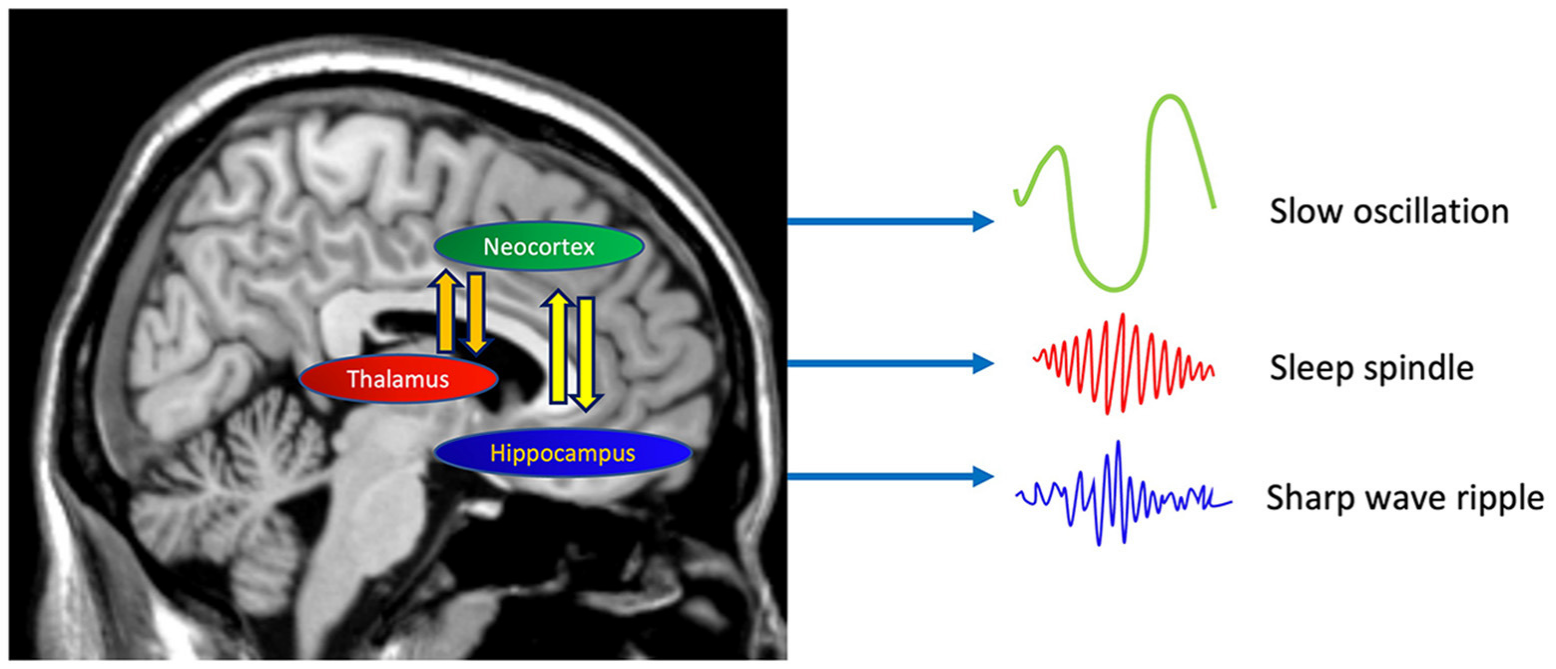
Neural oscillation regulating memory consolidation in NREM sleep. The oscillations of three different waves-slow oscillations from the neocortex, spindles from the thalamus, and ripples from the hippocampus are central to the induction of synaptic plasticity underlying memory consolidation during sleep.

One of the most striking examples of sleep microarchitectural analysis is the memory function of SO–spindle coupling. Communication between different brain networks relies on the interaction and coupling of various EEG signals and local field potential rhythms (Siegel et al., 2012). SWS is dominated by three types of oscillations: SOs, spindles, and hippocampal ripples. The anterior-to-posterior migration of SOs reaches subcortical structures, including the hippocampus, and thalamus-generated spindles spread through thalamocortical fibers to the neocortex and also reach the hippocampus. Several human studies have demonstrated that the timing or strength of SO–spindle coupling is crucial for the overnight consolidation of declarative (Helfrich et al., 2018) and non-declarative (Solano et al., 2021; Hahn et al., 2022) memories (Figure 4). Furthermore, as discussed in Section 3, research linking sleep microarchitecture to various diseases has become increasingly active in recent years. Growing evidence has revealed that SO–spindle coupling is impaired in older adults (Muehlroth et al., 2019) and in individuals with psychiatric disorders such as schizophrenia (Kozhemiako et al., 2022) and neurodevelopmental disorders (Mylonas et al., 2022). Low absolute delta power was significantly associated with incident hypertension, whereas no sleep macrostructural features were associated with it (Berger et al., 2022).

While the gold standard for sleep stage evaluation is visual inspection, automatic detection of microarchitectures such as SO and spindles is increasingly utilized, and various algorithms have been employed to detect SOs (Mölle et al., 2002; Esser et al., 2007; Staresina et al., 2015). Several open-source software/toolboxes are currently available for this purpose (Mensen et al., 2016; Djonlagic et al., 2021a). Nevertheless, no consensus on the criteria for SO has been established, and each study has used different definitions. The absolute number of detected SOs varies by definition, and as discussed later, the function of slow waves may differ depending on their frequency and amplitude, even if lumped into the same category of SWA. Therefore, referring to the definitions applied in each study is crucial to interpreting study results properly.

Sleep microarchitecture provides evidence of important dynamic characteristics of the sleep process that are not reflected in the conventional framework of macroarchitectural sleep evaluation. Although sleep staging is the basis of sleep medicine, it truncates finer details. Analyses of sleep microarchitecture provide potentially significant information regarding sleep.

#### 2.1.5 Functional connectivity during SWS

Functional connectivity in the field of neuroscience refers to the statistical dependence between the time series of brain activity quantified at various spatial scales, ranging from the single-neuron level to the brain region level (Friston, 1994). Although functional connectivity can be evaluated using both invasive and non-invasive methods, non-invasive imaging techniques such as EEG, magnetoencephalography (MEG), and functional magnetic resonance imaging (fMRI) have been increasingly employed. These techniques enable researchers to examine the integration and synchronization of the human brain during sleep, which is difficult to assess using conventional research methods.

An EEG–fMRI study has demonstrated a strong reduction in corticocortical connectivity in SWS, whereas a widespread increase in corticocortical connectivity was observed in N1 and N2 (Spoormaker et al., 2010). Other studies analyzing the blood oxygen level-dependent (BOLD) signal fluctuations measured with fMRI during sleep have reported a breakdown of intra-network connectivity in SWS, specifically in the correlation between the anterior and posterior nodes of the “default mode network” (Horovitz et al., 2009; Sämann et al., 2011; Boly et al., 2012). This breakdown of corticocortical connectivity in SWS is consistent with a finding from an EEG and transcranial magnetic stimulation (TMS) combined study during sleep, which reported that TMS-induced activation spread over the cortex during wakefulness but remained local in SWS (Massimini et al., 2005). These findings indicate that although SWS is characterized by the synchronization of cortical neurons at the microscopic level, it can also be accompanied with a loss of integration among specific cortical areas at the network level. Several studies utilizing a whole-brain model have also suggested that these connectivity changes during SWS can explain the transition from local to global SWS (Deco et al., 2014; Cakan et al., 2021).

Since the analysis of functional connectivity during sleep also provides information on the functional differences of the brains, applications of this technique include the detection of brain changes associated with aging and the development of biomarkers for psychiatric disorders (Leistedt et al., 2009; Hein et al., 2019).

### 2.2 Physiology of SWS/SWA

#### 2.2.1 Neural system and SWS/SWA

##### 2.2.1.1 Synaptic homeostasis and SWS/SWA

Synaptic homeostasis hypothesis (SHY) is an influential hypothesis regarding why sleep is necessary for organisms, and Tononi and Cirelli (2003, 2006) proposed that the role of SWS is to reduce synaptic strength potentiated by learning during wakefulness. The core of SHY is summarized in four points. (1) Wakefulness is associated with synaptic potentiation in cortical circuits. (2) Synaptic potentiation is linked to the homeostatic regulation of SWA (defined as activities of waves having 0.5–4.5-Hz frequencies in this context). (3) SWA is associated with synaptic downscaling. (4) Synaptic downscaling is associated with the beneficial effects of sleep on performance, particularly memory function.

During wakefulness, we continuously receive sensory stimuli from the outside world and process them to produce an output through thoughts or actions. Thus, as wakefulness persists, the intensity of the synapses used increases, resulting in an increase in excitability. However, an overall increase in the synaptic strength and excitability of neurons is undesirable, mainly in terms of energy consumption (Harris et al., 2012). Therefore, the overall synaptic strength needs to be reduced and maintained at a constant level through sleep (synaptic renormalization). SHY insists that “sleep is the price we have to pay for plasticity” (Tononi and Cirelli, 2006). Although indirectly, subsequent studies have provided results supporting the hypothesis. For example, rats exposed to a stimulating environment exhibit a widespread increase in SWA during subsequent sleep (Huber et al., 2007). This increase was positively correlated with the amount of time that the rats spent exploring their environment and with the cortical induction of brain-derived neurotrophic factor (BDNF), which is a marker of synaptic potentiation. Global decreases in the area of contact between cortical axon terminals and dendritic spines during sleep were observed in mice (De Vivo et al., 2017), and similar findings were also reported in *Drosophila melanogaster* (Bushey et al., 2011). In mice and rats, phosphorylation of AMPA receptors, a marker of synaptic strength, is higher during wakefulness and lower during sleep in the cerebral cortex (Vyazovskiy et al., 2008). In the rat frontal cortex, a correlation between sleep pressure and neural firing frequency throughout the sleep–wake cycle was identified (Vyazovskiy et al., 2009).

Meanwhile, several reports have suggested the upscaling of synapses during sleep. Studies with structural imaging have reported local increases in synapse numbers and the area of synaptic contact during sleep, as well as the emergence of new spines (Yang et al., 2014; Li et al., 2017). Another study reported that firing rates of cells in the visual cortex remain stable or increase during sleep after monocular deprivation (Frank, 2017).

As discussed in the next subsection, SHY provides a theoretical background for considering the role of SWS in memory consolidation and also highlights local aspects of SWS. Although SHY has not yet been fully proven (Frank, 2012; Timofeev and Chauvette, 2017), this hypothesis can integrate various findings regarding the function of SWA.

##### 2.2.1.2 Memory function and SWS/SWA

Observations indicating that sleep benefits memory have been present since the beginning of experimental memory research (Heine and Universit?t, 1914). The topic of discussion has revolved around the role of each sleep stage on memory. Recent research has highlighted the importance of SWS/SWA in memory consolidation, shifting the focus from the role of REM sleep in older research (Diekelmann and Born, 2010). Consolidation is the process by which recently encoded neuronal memory representations are reactivated and transformed into long-term memory during SWS (Stickgold, 2005).

Several key hypotheses have been proposed regarding how each sleep stage contributes to memory consolidation. (1) The dual-process hypothesis suggests that different stages of sleep consolidate different types of memories (Maquet, 2001; Rauchs et al., 2005). Specifically, it assumes that SWS benefits declarative memory consolidation, whereas REM sleep supports the consolidation of non-declarative memory. Although many human studies using the night-half paradigm support this hypothesis (Gais and Born, 2004), a major limitation of this paradigm is that it ignores the possible contributions of stage 2 sleep to memory. (2) The sequential hypothesis emphasizes the importance of the cyclic succession of non-REM and REM sleep in memory formation. The sleep stages may have complementary functions in this process. This hypothesis postulates that nonadaptive memories are weakened and adaptive responses are strengthened in the first processing step during SWS. In the second processing step during REM sleep, adaptive memories are integrated and stored in preexisting knowledge networks (Giuditta et al., 1995; Ambrosini and Giuditta, 2001). Many findings also support the sequential hypothesis; however, direct testing of this hypothesis is limited. (3) The active system consolidation hypothesis combines elements from both the dual-process view and sequential hypothesis. The core principle of this hypothesis is that sleep-dependent memory consolidation arises from the recurrent reactivation of newly formed memory representations during SWS. The oscillations of three different waves (microarchitectures)–SOs, spindles, and hippocampal ripples (triplet coupling)–are at the center of this theory (Figure 4) (Klinzing et al., 2019). The depolarizing phase of SOs induces the emergence of thalamic spindles, which may be linked to the efficient neural reactivations originating in hippocampal networks (Steriade, 2006; Staresina et al., 2015). The coupling between the SO and spindle is strengthened by prior learning, which is considered to boost synaptic plasticity in cortical pyramidal neurons (Niethard et al., 2018). Although the concept of an active redistribution of memory representations from temporary stores into long-term stores has been developed for hippocampus-dependent declarative memories, it might also hold for non-hippocampus-dependent memory.

Compared with the other hypotheses, active system consolidation not only differentiates the role of each sleep stage but also focuses more on sleep microarchitectures, including the SO and spindle, and integrates the neural mechanisms mediating the beneficial effect of sleep on memory. Several reasons explain why focusing on the sleep microarchitecture is crucial when investigating the memory function of slow waves. First, results regarding the involvement of slow waves in memory consolidation, especially in studies with older adults, have been inconsistent (Scullin and Bliwise, 2015). Some studies focusing on the role of SWS (macroarchitecture) in declarative memory consolidation in aging populations reported negative results (Seeck-Hirschner et al., 2012; Scullin, 2013). By contrast, the degree of impaired SWA (defined as 0.8–4.6 Hz) in older adults was reported to predict worse overnight memory consolidation (Mander et al., 2013). Furthermore, recent studies have reported an association between SO and memory consolidation by assessing coupling with spindle. (Helfrich et al., 2018; Mikutta et al., 2019; Muehlroth et al., 2019). Older adults exhibited reduced SO-spindle coupling, misalignment of the spindle, which occurred earlier in the rising flank of SO, and diminished overnight memory retention (Helfrich et al., 2018). The timing of this coupling is critical, as optimal memory consolidation has been reported to occur when spindles are aligned just after the peak of the SO up-state. Second, several studies have demonstrated that non-invasive brain stimulation applied to increase slow oscillatory activity during post-encoding SWS enhances the memory-promoting effects of sleep (Marshall et al., 2006) (See Section 4.3 for more detail). Third, several reports have suggested that even waves that have been lumped together as SWA may have different contributions to memory in different frequency bands. A study on rats has reported that SOs and delta waves, which are distinguished by distinct waveforms, have dissociable and competing roles in consolidation vs. forgetting (Kim et al., 2019). A human study on healthy older adults has reported that SO (0.5–1 Hz), but not a faster delta band (1–4 Hz) power, was positively associated with memory function (Kawai et al., 2021).

Research on sleep and memory has been advanced by focusing on brain rhythms. The current situation in which the definition of “slow wave” remains vague and mixed needs to be improved, and more rigorous classification and evaluation of slow waves in both animal and human studies are required for the future development of this field.

#### 2.2.2 Endocrine system and SWS/SWA

**Figure 5:**
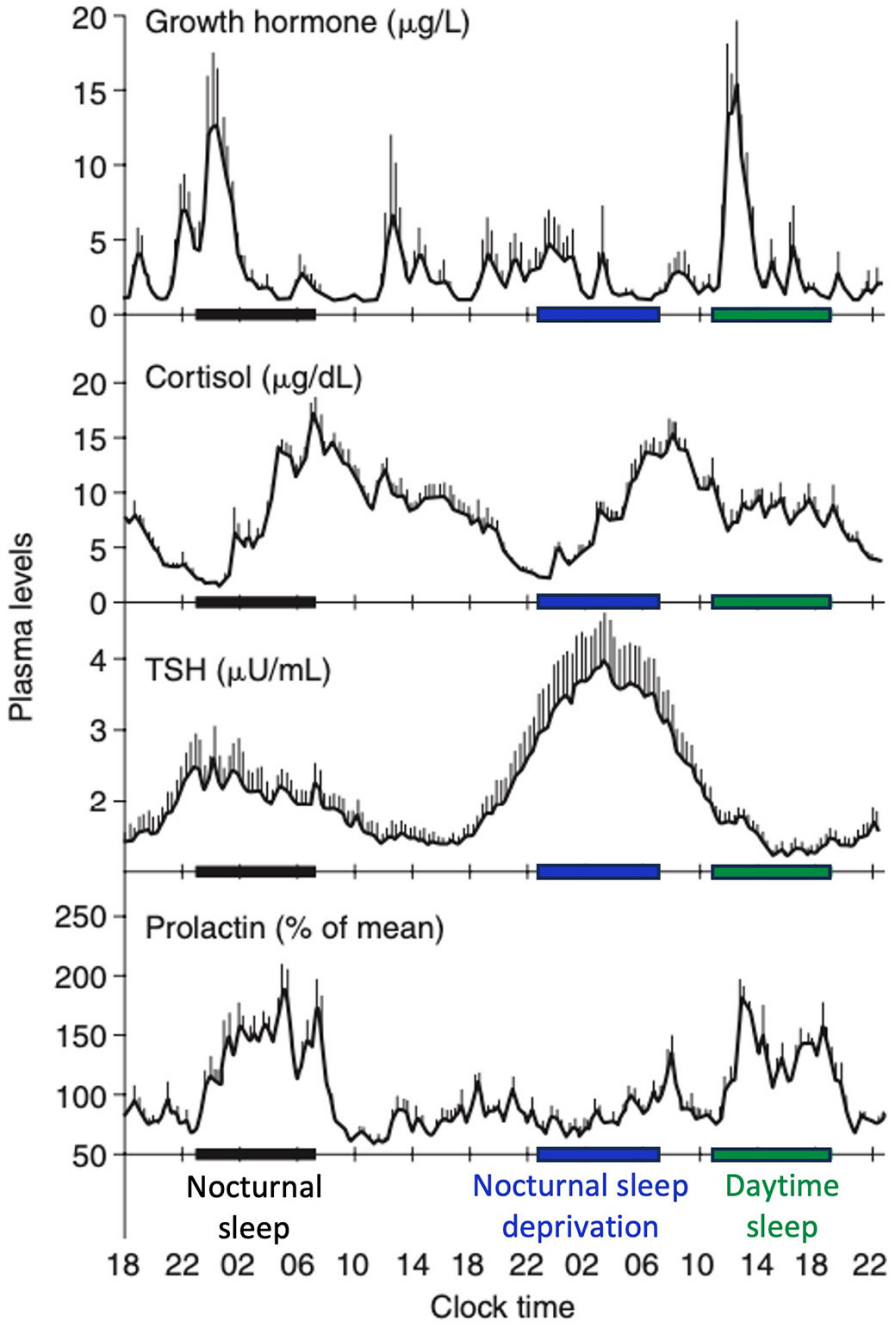
Contributions of the circadian rhythmicity and the sleep-wake homeostasis to hormone secretion. Shifted sleep immediately affects GH and PRL secretion, while cortisol and TSH secretion remain synchronized to circadian time. Modified from Van Cauter and Spiegel (1999) and Liu (2024).

SWS plays a key role in the regulation of various hormonal axes. Its impact can be dual, with both promoting and inhibitory effects, depending on the specific hormones involved (Figure 5).

A robust correlation has been identified between SWS and the secretion of growth hormone (GH), a sleep-stage-dependent hormone that is primarily produced during SWS (Takahashi et al., 1968), irrespective of age (Van Cauter et al., 2000). For individuals with isolated GH deficiency, SWS is diminished compared with healthy controls (Astrom and Jochumsen, 1989). However, the results may vary depending on the type of GH deficiency (pituitary vs. hypothalamic) (Copinschi et al., 2010). Previous studies have reported the conflicting effects of insulin-like growth factor-1 (IGF-1), a neurotrophic mediator of GH, on SWS (Prinz, 1995; Shah et al., 2013). The highest release of prolactin occurs after sleep onset, irrespective of the time of day. Although a close temporal association between SWA and increased PRL secretion is known (Spiegel et al., 1995), no quantitative correlation has been identified between SWA and PRL as seen between SWA and GH release in men. Meanwhile, increased SWS may be associated with higher prolactin levels, as observed in breastfeeding women with higher levels of SWS and delta power (Blyton et al., 2002; Nishihara et al., 2004). SWS exerts an inhibitory effect on the hypothalamic-pituitary-adrenal (HPA) axis, leading to elevated cortisol levels during the latter portion of the night, which coincides with reduced SWS and increased REM sleep (De Feijter et al., 2022). During puberty, the release of luteinizing hormone (LH) following sleep onset is positively associated with SWS (Shaw et al., 2012). LH levels tended to increase 15 min after the initiation of SWS, suggesting the potential influence of hypothalamic NREM-active neurons on gonadotropin-releasing hormone (GnRH) or other neurons responsible for GnRH secretion (Shaw et al., 2012). Thyroid-stimulating hormone secretion, regulated by the circadian rhythm, has been demonstrated to be negatively correlated with SWS (Shekhar et al., 2021). Regarding the renin-angiotensin-aldosteron system, higher plasma renin activity and aldosterone levels during SWS than during REM sleep have been reported (Brandenberger et al., 1994; Charloux et al., 1999).

In addition to the effects on hormones described so far, the impact of SWS on glucose and lipid metabolism has received increasing attention. A shorter SWS is associated with reduced insulin sensitivity and may be correlated with a higher incidence of type 2 diabetes (Kianersi et al., 2023). A decline in the SWS over time may be associated with weight gain (Van Cauter et al., 2008; Reither et al., 2021). These findings may be due to a combination of factors, including alterations in appetite-regulating hormones such as leptin, ghrelin, and GLP-1 via activation of the orexigenic system (for reviews, see Reutrakul and Van Cauter, 2018; Antza et al., 2021).

The physiological role of SWS on the endocrine system is diverse and underscores the multifaceted impact of SWS. Although the relationship between sleep and hormones has been known for a long time, it is still an “old and new” field full of unexplained details.

#### 2.2.3 Immune system and SWS/SWA

A bidirectional relationship between SWS and immunity has been known; activation of the immune system enhances SWS and increased SWS benefits the immune system.

Regarding the immune-to-SWS directionality, cytokines induced by the inflammatory response, such as interleukin (IL)-1β and tumor necrosis factor (TNF), are known to be involved in the regulation of SWS (Majde and Krueger, 2005). Administration of granulocyte colony-stimulating factor, which increases endogenous IL-1 antagonists and soluble TNF receptors, reduces SWS and delta power (Schuld et al., 1999). Intranasal administration of IL-6 has also been reported to enhance SWS/SWA in the second half of the night (Benedict et al., 2009). Meanwhile, other human studies have indicated that acute administration of IL-6 or interferon-α, which is reported to induce NREM sleep in animal studies, decreases SWS during the early part of the night (Späth-Schwalbe et al., 1998, 2000). More recent studies have suggested that the administration of anti-inflammatory drugs, such as an IL-1 receptor antagonist and tetracycline that suppresses TNF production, increases SWA (Schmidt et al., 2015; Besedovsky et al., 2017). As most of the knowledge regarding the effect of cytokines on sleep to date is from animal studies, these inconsistent results may be due to more complex pro- and anti-inflammatory effects in humans (Irwin, 2015). A few studies have reported the effect of commonly used nonsteroidal anti-inflammatory drugs on SWS. In healthy adults, acute administration of aspirin at the daily dose decreases SWS (Horne et al., 1980), and ibuprofen delays the onset of SWS (Murphy et al., 1994). These findings may be related to reduced prostaglandin synthesis, including decreases in prostaglandin D2, but evidence is still lacking.

As for the SWS-to-immune system directionality, the interaction between SWS and the endocrine system (Section 2.2.2) has been considered to play a crucial role. The amount of SWS and SWA during the night following hepatitis A vaccination has been reported to correlate with the percentage of antigen-specific CD4^+^ T (Th) cells measured up to 1 year later (Lange et al., 2011). Moreover, the accompanying increases in GH and prolactin and reduction in cortisol levels are also associated with this correlation. GH, prolactin, and aldosterone support the release of cytokines, cell proliferation, lymphocyte responses, and T-cell migration (Petrovsky, 2001; Kelley et al., 2007; Besedovsky et al., 2012). Low cortisol levels during SWS may enhance efficient antigen-presenting cells (APCs)-T cell interactions (Petrovsky, 2001; Glaser and Kiecolt-Glaser, 2005). Taken together, SWS may play a role in facilitating the interaction between APCs and T cells, which leads to a stronger immunological memory, by inducing the optimal pro-inflammatory “endocrine milieu” (Besedovsky et al., 2019).

As with memory and sleep research, the challenge regarding research on immune and sleep interaction is to assess the involvement of each sleep stage separately. However, increasing evidence supports the concept that immunological memory also consolidates during SWS as an analogy to the active systems consolidation hypothesis in memory research (Section 2.2.1.2) (Westermann et al., 2015). The interaction between SWS and immunity is deeply related to hormone secretion, and this can be viewed as a SWS-mediated neuro-immune-endocrine linkage. Although evidence in humans is still limited, this is an area expected to develop in the future.

#### 2.2.4 Feeling of rest, sleep inertia, and SWS/SWA

SWS plays a significant role in reducing sleepiness and aiding restoration, both subjectively and objectively. However, waking from SWS can cause residual sleepiness and sleep inertia. Understanding the connections among feelings of rest, sleepiness, sleep inertia, and SWS is crucial in the field of sleep physiology.

Reports have mainly associated a subjective feeling of being rested in the morning with higher sleep efficiency, longer total sleep time, and duration of SWS (Keklund and Akerstedt, 1997; Kohyama, 2021). SWS is the only sleep stage associated with subjective feelings of rest (Keklund and Akerstedt, 1997). Objectively, a higher SWS is crucial for physiological restorative sleep because of several factors such as a significant reduction in sympathetic activity, energy conservation, and an increase in growth hormone secretion that helps with the repair process. Disruption of the macro- and microarchitecture of the SWS, such as the occurrence of arousals or the presence of alpha intrusions (alpha–delta sleep), has been reported to be associated with unrefreshing sleep in various disorders, such as chronic fatigue syndrome, fibromyalgia, and some psychiatric disorders (Van Hoof et al., 2007; Vijayan et al., 2015; Wu et al., 2017).

Although the extent of SWS is closely linked to the subjective feeling of rest, awakening during SWS produces more sleep inertia than during other sleep stages (Tassi and Muzet, 2000). Sleep inertia is a transitional state characterized by reduced alertness and impaired cognitive performance upon awakening. It is characterized by grogginess, confusion, and a decline in cognitive and sensorimotor abilities (Hilditch and McHill, 2019). Individuals may also experience a decline in mood following awakening from SWS at night compared to their mood before sleep. Although the mechanism of sleep inertia is still unclear, the continuance of low-frequency brain activity in the minutes after awakening, particularly in the posterior regions of the brain, has been observed during sleep inertia (Ferrara et al., 2006; Marzano et al., 2011). Evidence supports a relationship between sleep inertia, intrusions of sleep-specific EEG characteristics, and sleep-specific brain functional connectivity. Compared to N2, waking up from SWS causes greater disruption of brain functional connectivity (Vallat et al., 2019). Global spectral power analysis after waking from the SWS revealed that the recovery of the global power of lower frequencies is more rapid than that of higher frequencies during sleep inertia. Moreover, the reduction in clustering and higher path length in the delta band was associated with increased sleepiness scales and higher lapses in a psychomotor vigilance test (Hilditch et al., 2023).

Several studies have confirmed the benefits of napping on mood and cognitive performance (Hsouna et al., 2019; Dutheil et al., 2021). However, longer naps are more inclined to transition from a lighter sleep stage to deeper sleep with a higher delta power, which is the main component of SWS. SWS is closely associated with sleep inertia, and can negatively affect short-term cognitive performance and alertness (Takahashi et al., 1998; Tietzel and Lack, 2001). Several studies have recommended limiting nap duration to 10–20 min to avoid this transition (Hayashi and Hori, 1998; Hayashi et al., 1999; Tietzel and Lack, 2002). Immediately after a 10-min nap, a notable increase in cognitive performance was observed, and this heightened state was maintained for at least an hour (Tietzel and Lack, 2001). By contrast, a 30-min nap in this study resulted in a decline in cognitive performance immediately after the nap, possibly because of sleep inertia. Interestingly, a study comparing 10-, 30-, and 60-min naps has reported that awakening during SWS was most prevalent in the 30-minute nap, despite an increased percentage of SWS with longer nap durations (Leong et al., 2023). However, this study also demonstrated that the memory encoding benefit is moderately associated with only a 30-min nap.

The link between higher SWS and feelings of rest is unique and has not yet been fully elucidated. Although longer nocturnal sleep may have desirable effects on restfulness, a limited nap duration is essential to maximize the benefits of naps (Tietzel and Lack, 2001; Fushimi and Hayashi, 2008). Exploring alterations in sleep microarchitecture, such as spectral power and functional connectivity, upon awakening from SWS can aid in elucidating the mechanisms underlying sleep inertia.

#### 2.2.5 Effects of sleep restriction on SWS

Sleep restriction, a highly effective treatment for chronic insomnia, typically involves an initial reduction in the amount of time spent in bed (Ong et al., 2016b). Over time, this intervention often leads to enhanced sleep efficiency and a gradual increase in the TST.

The impact of sleep restriction on various sleep stages has been studied, with a particular focus on SWS. Studies have revealed that reducing the time spent in bed to 4 h per night decreases the duration of sleep stages N1, N2, and REM, but not SWS, which is preserved (Brunner et al., 1990, 1993). In cases of sleep restriction lasting ~5 h per night, the N1, N2, and REM sleep stages tend to decrease, although the percentage of SWS often increases (Voderholzer et al., 2011). This change in SWS can persist even during the recovery phase when longer sleep durations are permitted (Xin et al., 2022). An increase in SWS and REM sleep may also occur during sleep recovery after sleep fragmentation or deprivation (Cheng et al., 2021). Delta power tended to increase initially with partial sleep deprivation. A direct relationship has been observed in rats, where shorter sleep durations correspond to greater increases in delta power (Kim et al., 2007). During sleep restriction in humans, the cumulative sum of delta power across each sleep period, referred to as slow wave energy, was lower than the baseline. However, the response during the recovery phase varied with sleep recovery duration (Banks et al., 2010). An increase in delta power was observed during both sleep restriction and recovery night, whereas the total slow-wave count increased only during the recovery night (Åkerstedt et al., 2009; Plante et al., 2016). A higher aggregate slow-wave amplitude in the frontal region occurs owing to a proportional increase in the quantity of high-amplitude slow waves, coupled with a simultaneous decrease in the count of low-amplitude slow waves from baseline to the sleep restriction and recovery periods. During the recovery period, the slopes of low-amplitude waves increase in the frontal region, but not of high-amplitude waves (Plante et al., 2016).

Although sleep restriction is a valuable tool for the management of chronic insomnia, its potential impact on sleep staging and microarchitecture needs to be considered. The adaptability of the SWS to sleep restriction highlights its significance in restorative processes. The dynamic changes in microarchitecture observed during sleep restriction and subsequent recovery contribute to a deeper understanding of sleep homeostasis.

## 3 Clinical science and practice of SWS and SWA

Given the significant physiological role of SWS, it would not be an exaggeration to say that SWS is involved in all disease and disorder processes. Thus, while more diseases need to be addressed in this regard in the future, knowledge of the relationship between SWS and disease is currently limited, with the majority of research focusing on neuropsychiatric disorders. Hence, we address neuropsychiatric disorders in the following three subsections (3.1–3.3). Although SDB has not necessarily been studied specifically with SWS, it is discussed in a separate subsection (3.4), considering its close association with other areas such as cognitive decline. Non-REM parasomnia related to SWS (3.5) and the issue of consciousness (3.6), which requires collaborative initiatives between research and clinical fields, are also addressed.

### 3.1 Aging, dementia, and SWS/SWA

Patients with Alzheimer's disease (AD) and other dementias have been known to have various sleep problems. Meanwhile, after a report that demonstrated amyloid-beta (Aβ) in the cerebrospinal fluid (CSF) is cleared during sleep (Kang et al., 2009), the bidirectional relationship between sleep disturbances and dementia has rapidly gained the interest of many researchers and clinicians. Subsequent reports have indicated that SWA is related to Aβ clearance (Ju et al., 2017).

From an anatomical perspective, a reduction in SWS has been associated with changes in the brain during aging. Loss of SWS is correlated with age-related frontal atrophy (Mander et al., 2013), gray matter reduction, and lower cortical/subcortical brain volume (Baril et al., 2021). In addition, a study using diffusion MRI has reported widespread correlations between various diffusion tensor-based metrics of white matter integrity and slow-wave slopes, indicating that reduced slow waves may be associated with white matter deterioration in older adults (Gudberg et al., 2022).

Reduced SWA generation has been associated with Aβ burden in the medial prefrontal cortex and impaired overnight memory consolidation (Mander et al., 2015). Changes in sleep begin before the clinical manifestations of dementia (Hudon et al., 2020). Individuals with cognitive complaints or those diagnosed with mild cognitive impairment (MCI) have decreased SWS compared with cognitively healthy individuals (Wei et al., 2024). Patients with AD have a significantly lower SWS percentage than controls (Zhang et al., 2022). However, longitudinal studies demonstrating a cause-effect relationship between SWS/SWA reduction and cognitive decline in the long term are still lacking (Song et al., 2015; Romanella et al., 2021). Meanwhile, higher slow-wave energy during non-REM sleep, which is a common marker of sleep need, in healthy young men has been associated with genetic liability for developing AD, as assessed by polygenic risk scores (Muto et al., 2021).

Further studies are needed to clarify the details of the causality between the decrease in SWA in MCI/AD and anatomical/functional changes in the brain, namely, whether the decrease in SWA is a “consequence” of the impairment of the frontal area, which may be the primary origin of SWA, or the decrease in SWA is a “cause” that results in functional impairment and atrophy by accelerating the accumulation of Aβ and other proteins. Nevertheless, many findings have indicated that complex interactions involving various factors, such as the circadian clock, apolipoprotein E, and sleep-disordered breathing (SDB), form a bidirectional relationship between SWA and dementia (Wang and Holtzman, 2019). The next point of interest on this topic is whether augmenting SWS can prevent the onset of AD (See Section 4.3).

#### 3.1.1 Glymphatic system: role of SWS in amyloid-beta and tau protein clearance

Studies on the glymphatic system have indicated its involvement in the pathogenesis of proteinopathies, including AD (Nedergaard and Goldman, 2020). The glymphatic system is a collaborative neural lymphatic network consisting of the circulation of CSF in the perivascular space through the astrocytic aquaporin-4 channel and lymphatic vessels located in the dura, covering both the brain and spinal cord (Iliff et al., 2012; Nedergaard, 2013). They work together to effectively clear toxic and nonfunctional waste materials from the brain, such as Aβ, tau, α-synuclein, and inflammatory cytokines.

The glymphatic system is considered active during SWS. The volume of CSF entering the brain parenchyma from the subarachnoid space and into the periarterial spaces may increase during SWS (Xie et al., 2013). A study with mice has demonstrated that glymphatic influx correlates positively with cortical delta power (Hablitz et al., 2019). A short SWS duration is correlated with enlargement of the perivascular space, representing impaired perivascular drainage (Baril et al., 2022). Existing research has suggested that the accumulation of Aβ, tau, and α-synuclein can lead to reduced glymphatic activity (Lopes et al., 2022). Several studies have reported a negative correlation between the amount of SWS and Aβ levels in CSF; further, the disruption of SWA during sleep resulted in increased CSF Aβ levels in individuals with normal cognitive function in several studies. However, conflicting data also exist (Ju et al., 2017; Sangalli and Boggero, 2023), and data indicating a causal relationship between SWS enhancement and AD biomarkers are lacking.

Although current data suggest that a higher SWS may contribute to the optimal glymphatic clearance of neurotoxic substances, data regarding the relationship between the glymphatic system and sleep characteristics in healthy adults remain limited (Sangalli and Boggero, 2023). Developments in this field may contribute to future dementia treatment.

### 3.2 Neurodevelopmental disorders and SWS/SWA

NDDs are a group of disorders that affect the growth, development, and function of the central nervous system. These disorders comprise various disabilities, including intellectual disorders, communication disorders, autism spectrum disorder, attention-deficit/hyperactivity disorder (ADHD), neurodevelopmental motor disorders, and specific learning disorders. As discussed in Section 2.1.3, the SWS changes drastically as the brain develops, and many studies have also reported that adequate SWS is crucial in healthy brain development (Ringli and Huber, 2011). As the proportion of SWS is a reliable indicator of gray matter maturation, it is plausible that alterations in the SWS may also reflect atypical patterns of neuronal development in NDDs.

Compared to typically developing children, children with autism spectrum disorder (ASD) have been reported to have higher SWS and less REM sleep (Buckley et al., 2010; Kawai et al., 2023), although several inconsistent reports also exist (Aathira et al., 2017; Lehoux et al., 2019). The disrupted SO-spindle coupling has been observed in ASD, which may be linked to the dysregulation of thalamocortical interactions (Mylonas et al., 2022).

SWA in a pediatric ADHD sample was notably higher during early childhood, but gradually decreased in late childhood and adolescence compared to a control group of healthy individuals (Biancardi et al., 2021). Regarding slow-wave architecture in ADHD, the maximal downward slopes of slow waves were positively correlated with the presence of multiple anxiety disorders and autistic features, while representing a negative correlation with the Child Behavioral Checklist score (Fasano et al., 2022).

SWS alterations in Rett syndrome, a genetic disorder characterized by the regression of language and motor disabilities, include increased SWS percentage, heightened delta power during SWS, and numerous SWS fragmentations (Spruyt, 2022).

Children with Angelman syndrome, a genetic disorder characterized by severe intellectual disability, happy demeanors, ataxia, seizures, and a reduced need for sleep, were observed to have a higher proportion of SWS (Laan and Vein, 2005). Moreover, they often display a distinctive pattern of notched rhythmic, high-amplitude delta activity (Korff et al., 2005).

Studies of children with Down syndrome, the most prevalent intellectual disorder due to genetic factors, have reported inconsistent results on SWS. While a significantly larger percentage of SWS in children aged two years and older has been reported in one study (Nisbet et al., 2015), more recent and larger studies did not identify a significant difference in the amount of SWS in children before school age when compared to children without Down syndrome (Kahanowitch et al., 2023). This latter finding has been reported in pediatric patients with Down syndrome, both with and without OSA (Heubi et al., 2021).

NDDs include a heterogeneous group of conditions with various genetic backgrounds and acquired factors, and the relationship between each disease and SWS also differs. In many NDDs, the patients have been reported to have a higher rate of sleep problems than typically developing individuals, suggesting an involvement of genetic factors. Meanwhile, further research is needed to determine the causal relationship between the presence of sleep problems and a degree of symptom progression, and whether intervention in SWS can alter the natural course of the disorders. Studying the association between NDDs and SWS is expected to contribute to the elucidation of the mechanisms of the disorders and open up possibilities for new interventions.

### 3.3 Psychiatric disorders and SWS/SWA

Increasing evidence has suggested a bidirectional relationship between many psychiatric disorders and sleep. It is no exaggeration to say that almost all psychiatric disorders can be associated with sleep problems, and clinical interest centers on whether intervention for sleep can improve the symptoms of disorders. Furthermore, an increasing number of studies have been attempting to link sleep microarchitecture to disease mechanisms and biomarker development.

A meta-analysis study showed that patients with schizophrenia had decreased SWS compared to healthy controls (Chan et al., 2017). Other studies have indicated that a reduction in SWS and an increase in REM latency are already present in the early phases of psychosis and might be the prodromal symptoms of the disorder (Zanini et al., 2013; Davies et al., 2017). The hypothesis that abnormalities in thalamocortical circuits underlie schizophrenia has led to the analysis of sleep microarchitecture, which may reflect thalamocortical/corticocortical circuit function (Castelnovo et al., 2018). A reduced SO count, density, and SWA in schizophrenia have been reported, although some inconsistent reports also exist, possibly due to the mixed definition of slow waves. Further, these alterations regarding slow waves, including decreased slow wave amplitude and slopes, were also observed in unaffected first-degree relatives of patients with schizophrenia (D'Agostino et al., 2018).

A reduction in SWS production, together with a decrease in REM latency, an increase in the total amount of REM sleep, and REM density have been reported in patients with major depressive disorder (MDD) (Armitage, 2007; Nutt et al., 2008; Pillai et al., 2011). Several studies with micro-architectural analysis have also showed reduced SWA throughout the night in MDD, although Armitage et al. reported that this was only observed in men (Borbély et al., 1984; Kupfer et al., 1989; Armitage et al., 2000). Another study has shown that lower delta wave counts in MDD are limited to the first NREM period of the night, which is related to the clinical course of the disease and symptom severity (Kupfer et al., 1990). These changes in the SWS percentage and REM density have also been observed in patients at a high risk of developing depression (Pillai et al., 2011). Power spectral analysis of EEG in different MDD subtypes revealed reduced absolute delta power only in the melancholic subtype of MDD compared to non-MDD (Solelhac et al., 2023). Although the data on SWS in bipolar disorder is limited, a meta-analysis study has revealed no significant alterations in the quantity or proportion of SWS in individuals with seasonal affective disorders (Baglioni et al., 2016).

SWS and sleep efficiency are lower in patients with post-traumatic stress disorder than in healthy controls (Kobayashi et al., 2007). Similarly, a smaller percentage of SWS was observed in adults with anxiety disorders than in those without anxiety (Fuller et al., 1997).

Disparate and inconsistent reports on SWS findings in psychiatric disorders exist. In addition to the problem of mixed slow wave definitions, the presence of multiple comorbidities in many psychiatric disorders also hinders the availability of reliable data. However, as in the case of SO-spindle analysis in schizophrenia, research on the relationship between sleep microarchitecture and the mechanisms of psychiatric disorders will become increasingly significant in the future.

### 3.4 SDB and SWS/SWA

Individuals diagnosed with SDB demonstrate significant sleep disruption, with obstructive sleep apnea (OSA) being the most prevalent form of SDB. OSA, characterized by intermittent partial or complete upper airway obstruction during sleep, is associated with an increased cardiovascular risk and poorer cognitive function. SWS alteration has been observed not only in OSA but also in other SDBs, such as central sleep apnea (CSA) and obesity hypoventilation syndrome (OHS).

Several studies have reported reduced SWS/SWA during NREM sleep (Himanen et al., 2004; Saunamäki et al., 2009; Jones et al., 2014) and increased delta power while awake in individuals with OSA (Morisson et al., 1998; Grenèche et al., 2008). However, seemingly inconsistent results also exist (for review, see D'Rozario et al., 2017). Higher delta power during NREM sleep has been observed in patients with severe OSA than in those with simple snoring or mild-moderate OSA (Kang et al., 2020; Liu et al., 2021). The delta/alpha power ratio has been reported to be higher in adults with OSA (Wang et al., 2021). Although inconclusive, these seemingly paradoxical results may be partly due to a compensatory EEG slowing response to arousal (Svanborg and Guilleminault, 1996; Huang et al., 2018) or precedence of slow waves over respiratory arousal in OSA (De Carli et al., 2004). Furthermore, a higher percentage of SWS has been observed in hypercapnic patients with OSA, suggesting the association between hypercapnia and EEG slowing (Wang et al., 2011).

Adenotonsillectomy, the first-line treatment for pre-pubescent individuals with OSA (Ben-Israel et al., 2011), and continuous positive airway pressure (CPAP), the gold standard therapy for adults with OSA, have been demonstrated to increase SWS (Heinzer et al., 2001; Djonlagic et al., 2021b). An increase in the delta power within the parietal region was observed after CPAP treatment in patients with moderate and severe OSA (D'Rozario et al., 2023). Meanwhile, a decrease in the delta/alpha ratio has been observed after CPAP treatment for OSA (Wang et al., 2021), and this was correlated with a decrease in daytime pCO2 and sleepiness in hypercapnic patients with SDB (Wang et al., 2014).

SWS plays a significant role in maintaining healthy respiratory function during sleep, particularly in conditions such as OSA with a low-arousal threshold endotype. SWS has a high arousal threshold, providing time for the upper airway muscles to respond adequately and counteract pharyngeal collapse (Saboisky et al., 2010). Therefore, higher SWS may help prevent or mitigate episodes of airway obstruction, although this mechanism may be impaired in patients with hypercapnic respiratory failure.

Evidence has supported an association between SWS and cognitive function in patients with OSA. In adults with moderate and severe OSA, an increase in delta power following CPAP treatment was correlated with improved overnight consolidation of procedural memory (D'Rozario et al., 2023). However, in a large multi-cohort study, the percentage of SWS was not associated with global cognition in patients (Pase et al., 2023). Data on the effect of SWA on psychomotor vigilance tests are controversial. SWS is also associated with cardiovascular function in patients with OSA. A lower percentage of SWS has been associated with a higher risk of hypertension in OSA patients of both sexes (Javaheri et al., 2018; Ren et al., 2020). A study on elderly men yielded comparable results (Fung et al., 2011). A decrease in SWS among individuals with severe OSA has been linked to two cerebrovascular biomarkers in imaging studies: the presence of white matter hyperintensities and a reduction in the fractional anisotropy of the genu of the corpus callosum (Carvalho et al., 2023).

In patients with mixed OSA and OHS, a condition with awake alveolar hypoventilation and morbid obesity, CPAP treatment increases SWS and reduces daytime sleepiness (Salord et al., 2013). Individuals with OHS have increased theta power during waking and delta/alpha ratios during sleep (Sivam et al., 2020). CSA, a condition characterized by the absence of airflow and respiratory effort, is frequently caused by heart failure, and its mechanism is often attributed to a high loop gain. A low percentage of SWS during sleep has been observed in adults with this condition (Roder et al., 2020). Similarly, among children with idiopathic CSA, a lower percentage of SWS was observed than in the control group (Gurbani et al., 2017). Nevertheless, the direction of change in the SWS after treatment is still controversial (Teschler et al., 2001; Roder et al., 2020). Congenital central alveolar hypoventilation (CCAH) is a rare syndrome characterized by autonomic nervous system and automatic respiratory disruption. SWS latency is shorter in participants with CCAH than in controls; however, the duration of SWS does not differ between participants with and without CCAH (Attali et al., 2017).

Examining the alterations in the macro-/microarchitectures of SWS in individuals with SDB is crucial, given its association with the severity parameters, potential implications for airway protection, and treatment outcomes.

### 3.5 Disorders of arousal and SWS/SWA

In most situations, higher SWS is associated with better health outcomes. However, disorders of arousal or NREM-related parasomnia can occur when awakening from NREM sleep, particularly during SWS. These conditions are undesirable experiences of sleep–wake state dissociation. Different subtypes of NREM-related parasomnia with confusional arousal have been identified, including sleepwalking, sleep terrors, sleep-related eating disorders, and sexsomnia (Schenck et al., 1998; Idir et al., 2022).

SWS can occasionally lead to problems owing to its higher arousal threshold (Busby et al., 1994). SWS is the stage in which awakening is least likely to occur. This is particularly evident in children (Busby et al., 1994). This characteristic of SWS explains its close association with NREM-related parasomnia, which can be aggravated by sleep deprivation. Confusional arousal is characterized by mental or behavioral confusion during arousal from sleep while in bed, in the absence of terror or ambulation, often with vocalizations and poor recall of events the following day (Howell, 2012). This condition is typically self-limiting, and often occurs during the first half of the night. Although this behavior is typically benign, patients can occasionally become aggressive and violent (Mainieri et al., 2023). Elevated long-range functional connectivity in high-frequency bands (alpha–beta) over the anteroposterior network prior to confusional arousal has been observed (Idir et al., 2022).

Sleepwalking typically starts with confusional arousal and is associated with complex behaviors outside bed. Compared to controls, sleepwalkers experience higher SWA during SWS, more interruptions in SWS, and slower SWA decline (Idir et al., 2022). Sleep terrors are commonly characterized by abrupt terror, loud scream, or cries with autonomic involvement, including tachycardia, mydriasis, and diaphoresis. In a study on sleep terrors and sleepwalking, high SWS fragmentation and high slow/mixed arousal indices were proposed as markers of disorders of arousal (Lopez et al., 2018).

Although disorders of arousal are more prevalent in children than in adults and are generally considered benign, when they occur in adults, especially sleepwalking, they may be associated with functional impairment, neurodegenerative disease, or even all-cause mortality (Lopez et al., 2013; Zhang et al., 2021, 2023).

Gaining insight into SWS changes in micro- and macroarchitectural aspects could elucidate the mechanisms of disorders of arousal. Despite being typically considered benign, the potential for serious consequences underscores the need for broader research to improve the diagnosis and treatment of disorders of arousal.

### 3.6 Consciousness and slow waves

The presence of high-amplitude delta oscillations has been linked to diminished consciousness or unconsciousness state in clinical settings for a long time. This is because delta oscillations/SOs are observed in various unconscious states, including SWS, anesthesia, and disorders that cause impaired consciousness, including coma and the vegetative state (Husain, 2006).

Cortical silence during the down-state of each slow-wave cycle has been considered a cause for unconscious states. However, some reports have indicated that high-amplitude delta oscillations are also observed during consciousness, especially in children with developmental disorders and epilepsy, such as Angelman syndrome, Rett syndrome, and mitochondrial diseases (see Section 3.2). This seemingly paradoxical phenomenon may be associated with the local aspect of SO/delta oscillation (Nir et al., 2011). Based on the findings that slow waves are local phenomena, a controversy has arisen regarding the frontal and posterior cortices' role in mediating consciousness (Gaskell et al., 2017; Mashour, 2018). A study using hdEEG has demonstrated that posterior delta power predicts the absence of dreaming both in REM and non-REM sleep (Siclari et al., 2017). Studies on propofol-induced anesthesia have suggested that anterior (but not posterior) delta activity is observed during consciousness following propofol administration (Sanders et al., 2018). This suggests that cortical down-states in anterior regions are not necessarily linked to unconsciousness. Another study using EEG source localization concluded that both anterior and posterior regions are disrupted at different levels of anesthesia-induced unconsciousness, leading to two empirically distinguishable states of unconsciousness (Stephen et al., 2020). Although these discussions are still inconclusive, they indicate that the local aspects of slow waves provide clues to the functional localization of the brain.

As typified by the Glasgow Coma Scale, which is often used in clinical practice, clinical assessment of consciousness is based on whether a person shows a voluntary response. Consciousness has not been the subject of science for a long time because it is something difficult to measure objectively. However, over the last few decades, several theories of consciousness, such as integrated information theory (IIT), have drawn a link between the complexity of brain dynamics and consciousness (Tononi, 2008; Oizumi et al., 2014). IIT explains that large-scale neuronal synchronization during SWS may diminish consciousness by reducing the informational content of the thalamocortical system (Tononi et al., 2016).

Although scientific research on consciousness has just begun, slow waves have been considered a key to elucidating its mechanisms. Research on consciousness and slow waves is an interdisciplinary field expected to develop in the future, encompassing a wide range of areas from sleep science to mechanisms of anesthesia and diseases that cause impaired consciousness.

## 4 Current limitations and future directions of SWS/SWA

### 4.1 Problems with the definition of slow wave

As mentioned throughout this paper, different definitions being used for SWA, SO, and delta waves are a critical issue in this field, where many researchers and clinicians are involved. The first step is for all the people involved in this area to share the problems that currently exist. Results from future studies should be compared with previous data with a careful review of different definitions in order to confirm reproducibility and obtain a unified view. We propose holding an international conference with a wide range of researchers and clinicians to discuss what should be done in the future regarding the definition of SWS based on the current situation.

### 4.2 Scoring limitations and evolution

The conventional “macroscopic” approach to sleep stage scoring often represents a superficial view of EEG characteristics in terms of their temporal, spatial, and frequency domains and can be prone to variability among scorers owing to subjective, visual-based criteria. Conventional sleep stage scoring methods may underestimate changes in sleep, especially age-related alterations in sleep (Schwarz et al., 2017). Although polysomnography (PSG) of older adults have multiple epochs containing SWA, they often do not meet the AASM amplitude criteria for the SWS stage. Consequently, these epochs are often scored as N2 stage, which may explain the absence of an association between SWS and cognition.

PSG utilizing manual sleep scoring is the gold standard for diagnosing sleep disorders; however, it is time-consuming and subject to inter- and intra-rater variabilities. Consequently, machine-learning-driven automated approaches have been developed as alternatives. A meta-analysis of the variation in manual PSG scoring revealed substantial inter-rater agreement for overall scoring, awake and REM, and moderate inter-rater reliability for stages N2 and N3, while for stage N1, inter-rater reliability was poor (Lee et al., 2022b). Automated sleep-stage scoring has been developed using extensive PSG datasets on which machine learning and deep learning have been trained. Automated scoring can provide high-quality results, with Cohen's kappa exceeding 0.80 and accuracy surpassing 90% (Gaiduk et al., 2023). Moreover, automatic scoring facilitates the evaluation of sleep microarchitectures, which are difficult to capture manually. Currently, expert review following the automated scoring is still necessary to avoid missing certain pathologies. The routine implementation of automated scoring could greatly benefit clinicians and researchers by saving time and increasing scoring reliability.

### 4.3 Interventions that impact SWS/SWA

In this review, we examined the important health implications of SWS/SWA and its disadvantages to health when deficient from both basic science and clinical perspectives. One of the directions that naturally follows is whether artificially increasing SWS is feasible and whether this can help prevent disease and promote good health. Many studies have investigated this issue. This section summarizes reports published to date for each method.

#### 4.3.1 Acoustic stimulation

The use of acoustic stimulation has been shown to boost SO (<1 Hz) and SWA and improve sleep-dependent memory retention in young adults (Ngo et al., 2015; Ong et al., 2016a, 2018; Leminen et al., 2017) and older adults (Papalambros et al., 2017). This procedure has also been reported to enhance SWA in individuals with MCI and improve performance in word-recall tasks, although data on its long-term benefits remain limited (Papalambros et al., 2019). A more recent pilot study has demonstrated that acoustic stimulation could increase SWS in adults with mild-to-moderate AD; however, its effect on cognitive function has yet to be proven (Van Den Bulcke et al., 2023).

#### 4.3.2 Transcranial electrical stimulation and TMS

Transcranial direct current stimulation (tDCS) improves SWA and overnight memory retention in young adults (Marshall et al., 2004, 2006). Many studies have demonstrated an association between enhanced SO using tDCS and improved declarative memory in young and older adults, specifically for word-pair learning (Westerberg et al., 2015; Barham et al., 2016) and visuospatial tasks (Ladenbauer et al., 2016). However, there have also been some inconsistent results showing no enhanced SO or improved memory (Eggert et al., 2013; Paßmann et al., 2016; Bueno-Lopez et al., 2019). One meta-analysis has reported that SO augmentation did not enhance procedural memory (Barham et al., 2016). In individuals with MCI, an increase in SO has been observed with the improvement in visual declarative memory, but not with verbal memory improvement (Ladenbauer et al., 2017).

A few studies applying transcranial alternating current stimulation (tACS) have demonstrated an increase in SO and a dose–effect relationship (Jones et al., 2018; Ketz et al., 2018). Moreover, TMS applied during NREM sleep has been reported to augment SWS (Massimini et al., 2007). Although the effect of TMS may be more spatially precise than that of acoustic stimulation or tDCS, limited studies have been conducted to date (Feher et al., 2021).

In sum, many studies have succeeded in augmenting SWS using these techniques; however, there are mixed results concerning their beneficial effects on memory, especially in older adults. Further studies examining the long-term effects of augmenting SWS and its impact on the development of cognitive impairment are warranted.

#### 4.3.3 Pharmacological methods

Several pharmacotherapies have been discovered that extend the duration of SWS through different mechanisms, such as GABA_A_ agonist, GABA_B_/gamma hydroxybutyrate (GHB) agonist, serotonin-2A receptor antagonists (e.g., ritanserin and eplivanserin), and multiple receptor antagonists (e.g., trazodone and mirtazapine) (Walsh, 2009). Medications acting on α2-δ calcium channel ligands (e.g., pregabalin and gabapentin) have also been shown to enhance SWS. A study using Tiagabine, an inhibitor of GABA transporter 1, has demonstrated increased SWS and improved performance on cognitive tasks during sleep restriction (Walsh et al., 2006). However, another study with Tiagabine has reported no benefit of augmented SWS on memory consolidation in healthy adults (Feld et al., 2013). Sodium oxybate has also been associated with increased SWA and better cognition in healthy young adults (Hall, 2009; Walsh et al., 2010). Olanzapine, a serotonin-2A-dopamine-D3 receptor antagonist, similarly increased SWS in adults with schizophrenia (Göder et al., 2008) but decreased SWS latency without increasing SWS in adults with depression (Lazowski et al., 2014).

Meanwhile, several pharmacological interventions have been reported to decrease SWS. While antiepileptics that act on voltage-gated sodium channels, such as valproic acid and phenytoin, exhibit conflicting effects on SWS, phenobarbital, which prolongs the opening time of chloride channels by acting on GABA_A_ receptor subunits, typically appears to attenuate SWS (Carvalho et al., 2022). Similarly, benzodiazepines, a GABA_A_ receptor agonist, have also been associated with reduced SWS (De Mendonca et al., 2023). Although sex hormones might impact SWS, and women generally have higher SWS, no significant improvement in SWS was observed after gender-affirming hormone therapy (e.g., estrogen and antiandrogen) in the transfeminine group. However, SWS reduction after testosterone administration has been observed in the transmasculine group (Morssinkhof et al., 2023).

The benefits and disadvantages of drug-induced changes in SWS to human health have not been fully explored to date due to difficulties in the study design.

#### 4.3.4 Other methods

Exercise enhances SWS in both young and older adults (Cassim et al., 2022). When compared to the absence of exercise, acute evening moderate-intensity exercise had the highest ranking for improving the proportion of SWS, followed by high-intensity exercise and low-intensity exercise. Comparing acute evening moderate- and high-intensity exercise to low-intensity exercise, both interventions led to an increase in the proportion of SWS, and no significant difference was observed (Yue et al., 2022). Listening to the suggestion to “sleep deeper” while falling asleep can promote SWS and SWA (Cordi et al., 2014; Besedovsky et al., 2022). However, no improvement in declarative memory was observed with this intervention, which could be due to the lack of SO-spindle coupling (Beck et al., 2021).

## 5 Conclusion

This review summarizes existing reports on the health implications of slow waves from both the macro- and micro-perspectives. Currently, research is focused not only on elucidating the mechanisms underlying SWS, which remain a mystery, but also on whether interventions for SWS can contribute to better health. Nowadays, people are more concerned than ever about why we sleep and what good sleep is. For further development of this field, we re-emphasize the importance of being aware of mixed criteria regarding “slow wave.” Examining the definition of SWS from both the macro- and micro-perspectives in each study and sharing the understanding with those involved in this field are critical steps to move forward.

## Author contributions

TI: Conceptualization, Writing—review & editing, Writing—original draft, Visualization. PT: Writing—original draft, Writing— review & editing, Conceptualization. CC: Writing—review & editing. RO'H: Writing—review & editing, Conceptualization, Supervision. MK: Writing—original draft, Writing—review & editing, Conceptualization, Supervision.
